# Targeting of Proteoglycan Synthesis Pathway: A New Strategy to Counteract Excessive Matrix Proteoglycan Deposition and Transforming Growth Factor-β1-Induced Fibrotic Phenotype in Lung Fibroblasts

**DOI:** 10.1371/journal.pone.0146499

**Published:** 2016-01-11

**Authors:** Irfan Shaukat, Lydia Barré, Narayanan Venkatesan, Dong Li, Jean-Claude Jaquinet, Sylvie Fournel-Gigleux, Mohamed Ouzzine

**Affiliations:** 1 UMR 7365 CNRS-Université de Lorraine, Biopôle-Faculté de Médecine, CS 50184, 54505, Vandoeuvre-lès-Nancy, Cedex, France; 2 UMR 7311 CNRS-Institut de Chimie Organique et Analytique, Université d'Orléans-Pôle de Chimie, Rue de Chartres, 45067, Orléans, Cedex 02, France; University of Allabama at Birmingham, UNITED STATES

## Abstract

Stimulation of proteoglycan (PG) synthesis and deposition plays an important role in the pathophysiology of fibrosis and is an early and dominant feature of pulmonary fibrosis. Transforming growth factor-β1 (TGF-β1) is a major cytokine associated with fibrosis that induces excessive synthesis of matrix proteins, particularly PGs. Owing to the importance of PGs in matrix assembly and in mediating cytokine and growth factor signaling, a strategy based on the inhibition of PG synthesis may prevent excessive matrix PG deposition and attenuates profibrotic effects of TGF-β1 in lung fibroblasts. Here, we showed that 4-MU4-deoxy-β-D-xylopyranoside, a competitive inhibitor of β4-galactosyltransferase7, inhibited PG synthesis and secretion in a dose-dependent manner by decreasing the level of both chondroitin/dermatan- and heparin-sulfate PG in primary lung fibroblasts. Importantly, 4-MU4-deoxy-xyloside was able to counteract TGF-β1-induced synthesis of PGs, activation of fibroblast proliferation and fibroblast-myofibroblast differentiation. Mechanistically, 4-MU4-deoxy-xyloside treatment inhibited TGF-β1-induced activation of canonical Smads2/3 signaling pathway in lung primary fibroblasts. The knockdown of β4-galactosyltransferase7 mimicked 4-MU4-deoxy-xyloside effects, indicating selective inhibition of β4-galactosyltransferase7 by this compound. Collectively, this study reveals the anti-fibrotic activity of 4-MU4-deoxy-xyloside and indicates that inhibition of PG synthesis represents a novel strategy for the treatment of lung fibrosis.

## Introduction

Pulmonary fibrosis is characterized by injury and loss of lung epithelial cells, abnormal accumulation of myofibroblasts, and excessive deposition of collagen and proteoglycans (PGs) in the extracellular matrix (ECM), resulting in a progressive loss of pulmonary function [1]. However, the pathological basis of fibrosis is not completely understood. Recent studies have shown that abnormal regulation of PGs plays an important role in the pathophysiology of fibrosis and is an early and dominant features of fibrosis [2]. Indeed, abnormal accumulation of PGs has been shown to occur in pulmonary fibrosis in both human and animal models [3, 4], and is a part of the exacerbated accumulation of ECM constituents during the fibrotic process. In addition, it has been reported that the synthesis of both the core protein and glycosaminoglycan (GAG) chain was altered during the development of fibrosis. A consistent finding in animal models of lung fibrosis is an increase in the synthesis of chondroitin-sulfate/dermatan-sulfate (CS/DS) GAGs associated with accumulation of versican, a large CS-containing PG that forms macromolecular aggregates with hyaluronic acid in the interstitial matrix, and of decorin, which plays a key role in regulating collagen fibril formation and the spatial arrangement of collagen fibers in the matrix [4]. Increased accumulation of versican and decorin has been also reported in patients with pulmonary fibrosis [5]. In parallel, increased accumulation of heparan-sulfate (HS) PGs such as syndecan 1 are also observed in bleomyin-induced lung fibrosis [6] and in human idiopathic pulmonary fibrosis (IPF) [7].

In contrast to the detailed profiling of PG core protein expression in the fibrotic lung, changes in GAG structure and composition have begun only recently to be explored in detail. GAG chains are responsible for many of the biological properties and functions of PGs such as matrix deposition, intracellular signaling, morphogenesis, cell proliferation and migration [8]. Therefore, alterations in the synthesis of GAGs may contribute to disease development in fibrosis. In line with this, our recent study identified increased GAG content during tissue repair in fibrosis [9].

It is widely accepted that TGF-β is the major cytokine associated with pulmonary fibrosis [10] and is able to induce the synthesis of collagens and PGs [11]. Indeed, we showed recently that TGF-β1 increased PG synthesis in rat lung fibrobalasts by inducing the expression of XT-I (xylosyltransferase I) and GlcAT-I (β1, 3-glucuronyltransferase I), which regulate the rate of the PG synthesis [9]. These enzymes in addition to β4Galactosyltransferase7 (β4GalT7) and β3Galactosyltransferase6 (β3GalT6) are responsible for the initiation of the synthesis of GAG chains by catalysing the formation of the linkage tetrasaccharide (GlcAβ1, 3Galβ1, 3Galβ1, 4Xylβ1-O-Ser) that attaches the GAG chain to the PG core protein. Therefore, inhibition of any of the enzymes involved in these early steps of GAG chains synthesis such as β4GalT7 may provide a new strategy to prevent excess PG synthesis and deposition in fibrosis. In addition, owing to the importance of GAG chains of PGs in mediating cytokine and growth factor signaling, this strategy may attenuates TGF-β signaling and the associated profibrotic effects. Interestingly, xyloside analogues such as 4-Methylumbelliferyl-β-D-xylopyranoside can function as GAG chain initiators without a core protein. They are processed by β4GalT7 which adds a galactose residue on the hydroxyl group at C4 position of xylose followed by addition of a second galactose residue by β3GalT6 and of a glucuronic acid moiety by GlcAT-I to complete the tetrasaccharide primer before subsequent polymerisation of the GAG chain by other enzymes of the GAG synthetic pathway [12]. Taking advantage from the properties of these xylose analogues, we synthesized a competitive inhibitor of β4GalT7, 4-Methylumbelliferyl 4-deoxy-β-D-xylopyranoside (referred hereafter as 4-MU4-deoxy-xyloside) an analogue of 4-Methylumbelliferyl-β-D-xylopyranoside (4-MU-xyloside) that lacks the hydroxyl acceptor group at the C4 position of the xylose residue, required for the attachment of galactose by β4GalT7. Therefore, 4-MU4-deoxy-xyloside can bind to β4GalT7 but cannot be processed as in the case of 4-MU-xyloside. Here, we determined the ability of 4-MU4-deoxy-xyloside to reduce the synthesis and deposition of PGs in primary rat lung fibroblasts both in normal conditions and under TGF-β stimulation. We showed that 4-MU4-deoxy-xyloside inhibited PG synthesis and counteracted TGF-β1-induced PG synthesis in lung fibroblasts. We further demonstrated that 4-MU4-deoxy-xyloside antagonized TGF-β1 canonical signaling and reduced fibroblast proliferation. In addition, we bring evidence that 4-MU4-deoxy-xyloside prevented apparent fibroblast-to-myofibroblast trans-differentiation induced by TGF-β1, suggesting that inhibition of PG-GAG synthesis may provide a new therapeutic approach for IPF treatment.

## Materials and Methods

### Chemical and reagents

Cell culture medium (Dulbecco’s modified Eagle’s medium (DMEM)), Fetal Bovine Serum (FBS), Penicillin-Streptomycin, Glutamine, Phosphate Buffered Saline (PBS) were from Life Technology. Recombinant human TGF-β1 was from R&D Systems. Total and active TGF-β1 ELISA kit was from BioLegend. Na_2_[^35^S]SO_4_ was from Perkin Elmer and 4-Methylumbelliferyl-β-D-xylopyranoside (4-MU-xyloside) from Sigma-Aldrich.

### Synthesis of the 4-MU4-deoxy xyloside

4-Methylumbelliferyl 2, 3-di-O-benzoyl-β-D-xylopyranosid was prepared from 4-methylumbelliferyl β-D-xylopyranoside as previously reported [13] and 4-MU4-deoxy-xyloside was then prepared from the latter by radical deoxygenation followed by deprotection. The synthesized 4-MU4-deoxy-xyloside was able to inhibit β4GALT7 *in vitro* with an IC_50_ value of 1 mM [14].

### Ethics Statement

Rats were acclimated for 1 week in the laboratory before use. Animals were housed in groups of three or four in solid-bottomed plastic cages with free access to tap water and food. Room temperature was set at 23 ± 1°C and animals were subjected to a 12 h light cycle (with light on from 06:00 to 18:00). All animal experiments were conducted according to the recommendations of European Directive 2010/63/UE and French legislation article R.214-88.

### Cell culture and isolation

Primary rat lung fibroblasts were isolated as described before [15]. Briefly, rats were anesthetized by halothane then sacrificed by cervical dislocation to remove the lungs. The lungs were perfused by PBS in sterile conditions and minced into 2–3 mm pieces then digested in DMEM containing trypsin (2.5 mg/ml), collagenase (1 mg/ml), and DNase I (2 mg/ml) at 37°C with gentle stirring for 30 min. Lung cells were separated from undigested tissue and debris by centrifugation and washed twice with PBS. The cells were suspended in DMEM (4.5 mg/ml glucose) medium supplemented with 2 mM glutamin, 100 IU/ml penicillin, 100 μg/ml streptomycin and 10% FBS and grown in a culture plate at 37°C with humidified atmosphere in a 5% CO_2_. After 24 h, the medium was replaced to remove the unattached cells. When the cells were confluent, they were trypsinized and amplified. The cells formed homogeneous monolayers morphologically consistent with fibroblast-like cells.

### DNA transfection and luciferase assay

Lung fibroblasts (5x10^6^ cells/ml in suspension) were co-transfected with 2 μg of the p(CAGA)_12_-Lux reporter plasmid [16] and 100 ng of pRL-Tk plasmid encoding *Renilla luciferase*, used as internal control, by nucleofection using Lonza Primary Fibroblasts Transfection Kit (Lonza) according to manufacturer’s instructions. Cells were then seeded in 12-well plate at 1x10^5^ Cell/well. At 24 h post-transfection, cells were treated with 4-MU4-deoxy-xyloside and/or TGF-β1 (5 ng/ml) for 12 h in serum free medium as indicated in the figure legends. The luciferase activity was measured using a Dual Luciferase Reporter Assay System (Promega, Madison, WI, USA) and a Berthold luminometer (Bad Wildbad, Germany). p(CAGA)_12_-Lux reporter luciferase activity was normalized to pRL-TK vector activity and was expressed relative to the basal activity of empty pGL3Basic vector.

### SiRNA transfection

Lung fibroblasts were seeded in 6-well culture plate and grown for 24 h before transfection. Cells were transfected with the siRNA (25 nM) for β4GalT7 (GCCUGAACACUGUGAGGUA) (Sigma-aldrich) or siRNA control (Qiagen) using the DharmaFECT transfection reagent (Thermo Scientific, Waltham, MA) according to manufacturer's instructions. At 48 h post-transfection, cells were treated or not with TGF-β1 (5 ng/ml) for 3 h. The expression level of β4GalT7, αSMA, collagen I and TGF-β1 genes was analyzed by quantitative real-time PCR.

### Quantitative real-time PCR

After treatment, total RNA was extracted from lung fibroblasts by the RNAeasey Kit (Qiagen), according to manufacturer's instructions. The cDNAs were synthesized from 500 ng of total RNA using iScript reverse transcription supermix (Bio-Rad). cDNAs were subjected to quantitative PCR using iTaq Universal SYBR Green Supermix. (Bio-Rad). The expression of β4GalT7, αSMA, collagen I and TGF-β1 was measured using specific primers (Qiagen). PCR cycling parameters were 30 s at 95°C; 40 cycles of 30 s at 95°C and 60 s at 60°C. Expression levels of target genes were normalized to ribosomal protein S29 RNA level.

### Proteoglycan synthesis

Proteoglycan synthesis was measured by ^35^S-sulfate incorporation as described by De Vries et al., [17]. Briefly, fibroblasts were grown in 6-well culture plate until 80% confluence then treated with either 4-MU4-deoxy-xyloside or 4-MU-xyloside in the absence or presence of TGF-β1 (5 ng/ml) for 24 h. At 6 h before the end of the treatment, cells were radiolabeled with 10 μCi/ml of ^35^S-sulfate (Perkin Elmer, Courtabœuf, France). Culture medium was collected, digested with papain (1 mg/ml) and ^35^S-labeled GAGs were precipitated by cetylpyridinium chloride (CPC), dissolved in solvable and mixed in scintillation fluid (Perkin Elmer, MA, USA). The radioactivity associated with GAGs was measured by liquid scintillation counting (Packard, Rungis, France).

To analyze endogenous PG-GAG chains or GAG chains primed with 4-MU-xyloside, cells were metabolically labelled with 10 μCi/ml of ^35^S-sulfate (Perkin Elmer, Courtabœuf, France) for 24 h in the presence or not of 4-MU4-deoxy-xyloside. Culture medium was collected, digested with papain (1 mg/ml) and ^35^S-labeled GAGs were precipitated by CPC. Isolated GAGs were analyzed by SDS-PAGE in 4–12% Nu-PAGE gel. After migration the gel was dried and exposed to autoradiography film. The radioactivity corresponding to GAGs was normalized with the amount of DNA of corresponding cells.

### Cellular proliferation assay

The cell proliferation was measured by the CFSE incorporation as previously described [18]. Briefly, fibroblasts were trypsinized and washed twice with PBS then labelled with CFSE using CellTrace^™^ CFSE Cell Proliferation Kit (Life technologies) according to manufacturer’s instructions. Cells were then seeded onto a 12-well culture plate at 1x10^5^ cells/well and treated with 4-MU4-deoxy-xyloside and/or TGF-β1 (5 ng/ml) for 48 h. After treatment, the cells were analyzed on a Gallios FACS flow cytometer (Beckman coulter).

### Cell viability assay

Fibroblast cells were seeded in a 96-well culture plate and incubated with 4-MU4-deoxy-xyloside in the presence or absence of TGF-β1 (5 ng/ml) for 24 h. Cells were then exposed to MTT for 3 h and lysed with the addition of 100 μl of DMSO. Subsequently, the cell viability was determined by measuring the absorbance at 550 nm using the microplate spectrophotometer Varioskan Flash Multimode reader (Fisher Scientific).

### Western blotting

Fibroblasts were seeded in a 6-well culture plate and treated as indicated in the figure legends. The cells were then lysed in RIPA buffer and protein concentration was determined by Bradford method [19]. 30 μg of total protein was separated by SDS-PAGE and transferred onto the PVDF immobilon membrane (Millipore) then incubated overnight at 4°C with appropriate primary antibodies: anti-decorin (1:1000, R&D Systems), phospho-Smad2, phospho-Smad3 and Smad2/3 (1:1000, Cell Signaling Technology), αSMA (1: 5000, Sigma) and β-actin (1: 6000, Sigma-Aldrich) used as loading control. The protein bands were visualized by chemiluminescence using chemiluminescence luminol reagent (Bio-Rad). Densitometry analysis was performed using Image J software (version 1.46r, National Institute of health, Bethesda, MD, USA).

### Immunofluorescence

Fibroblasts were grown on cover slips and treated or not with 4-MU4-deoxy-xyloside for 24 h. The cells were fixed in 4% paraformaldehyde then stained with the 10E4 monoclonal anti-HS antibodies (AMSBIO) and anti-mouse IgG coupled with Alexa Fluor 488 (Molecular Probes, Invitrogen). The nuclei were stained with Hoechst 33342 (Molecular Probes-Invitrogen). Representative micrographs were observed using inverted microscope Leica DMI3000 B (Leica Microsystems).

### Quantification of active and total TGF-β1

Lung fibroblasts grown in 6-well culture plates were treated in serum free medium with 4-MU4-deoxy-xyloside and/or TGF-β1 (5 ng/ml) for 24 h. Then, cell culture supernatants were collected and both total and active TGF-β1 were quantified using LEGEND MAX™ Free Active TGF-β1 ELISA Kit according to manufacturer’s instructions. Briefly, 50 μl of cell culture supernatants were transferred to the anti-TGF-β1 pre-coated 96-well plate in triplicate and incubated at room temperature for 2 h. Then, each well was washed and incubated with 100 μl TGF-β1 detection antibody solution for 1 h. Next, the wells were washed and incubated with 100 μl Avidin-HRP for 30 min. After washing, the plate was incubated with 100 μl of substrate solution for 10 min. Then, the reaction was stopped by addition of 100 μl of stop solution to each well and the absorbance was measured at 450 nm with the microplate spectrophotometer, Varioskan Flash Multimode reader (Fisher Scientific). The concentration of TGF-β1 was calculated using a standard curve. To assess the total amount of TGF-β1, acid activation was performed to release free active TGF-β1 before processing as indicated above.

### Statistical analysis

The results are presented as mean ± SD of three independent experiments. Statistical differences between control and treated groups were evaluated using Student’s test. A two-sided P-value <0.05 was considered statistically significant for all analyses.

## Results

### 4-MU4-deoxy-xyloside inhibits PG synthesis in lung fibroblasts

**Fig 1:**
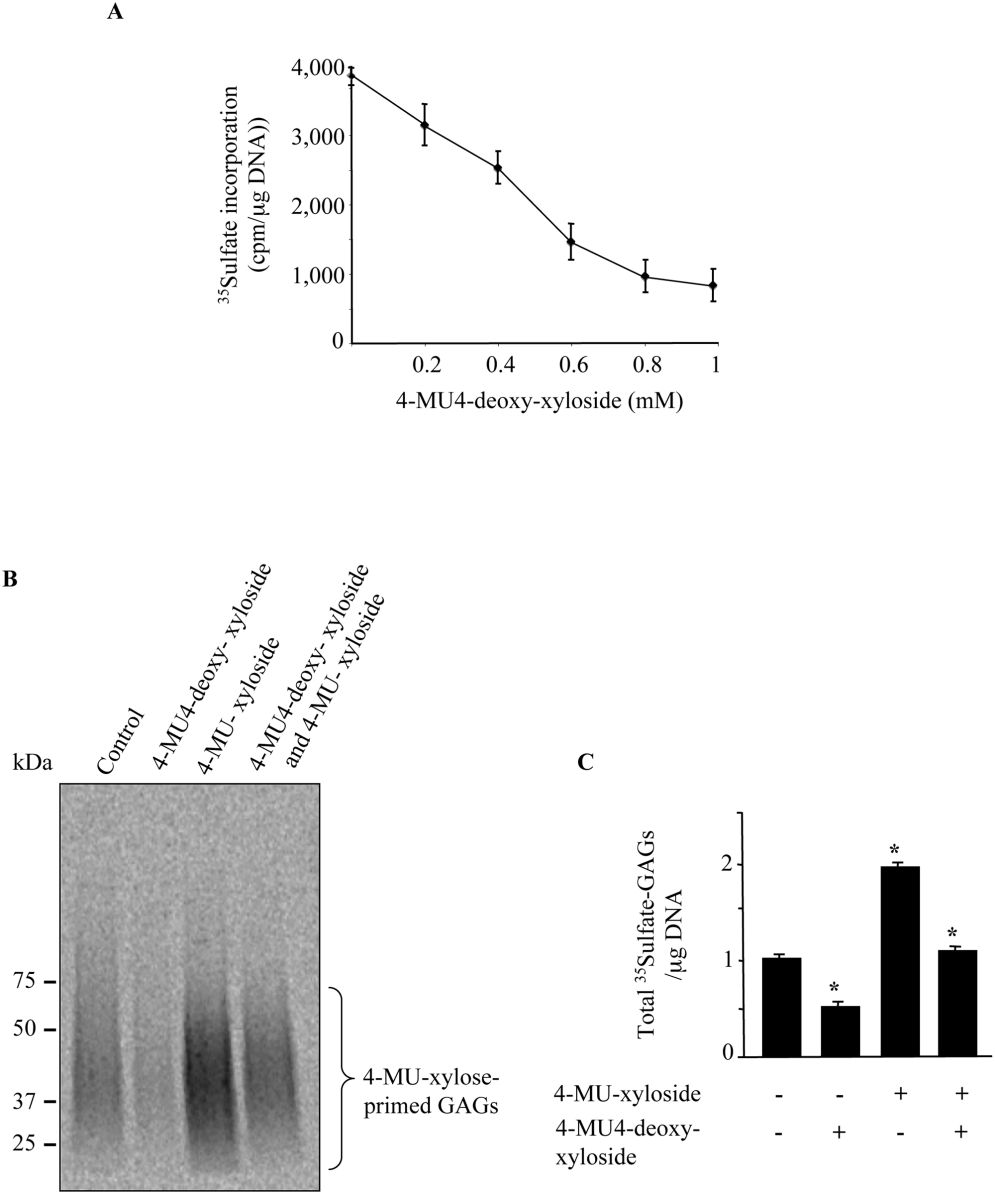
4-MU4-deoxy-xyloside inhibits PG synthesis. (**A**) Primary rat lung fibroblasts were treated with various concentrations of 4-MU4-deoxy-xyloside for 24 h and the level of the PG synthesis was measured by ^35^Sulfate incorporation for the last 6 h of the treatment. The control was treated with vehicle (DMSO). PG synthesis level was normalized to the amount of DNA. (**B**) Lung primary fibroblasts were incubated with or without 100 μM of 4-MU-xyloside in the presence and absence of 4-MU4-deoxy-xyloside (500 μM) and GAGs were metabolically labelled for 24 h with ^35^Sulfate incorporation. Radiolabeled GAGs were isolated from the medium by CPC precipitation and analyzed by SDS-PAGE then visualized by autoradiography. Bar graph shows total ^35^Sulfated GAGs normalized to DNA and relative to control (untreated). Data are expressed as mean ± SD of three separate experiments. *P<0.05 versus control.

With regard to the high level of PG synthesis and deposition in fibrosis, the development of strategies designed to tamper with PG production is clearly an attractive prospect for therapeutic application. Therefore, we investigated the ability of 4-MU4-deoxy-xyloside to inhibit PG synthesis in primary lung fibroblasts. We first analyzed the effect of 4-MU4-deoxy-xyloside on PG synthesis using ^35^S-Sulfation incorporation to label total GAG chains of PGs. Lung primary fibroblast cells were grown in medium containing radioactive Na_2_^35^SO_4_ in the absence (control) or presence of various concentrations of 4-MU4-deoxy-xyloside. The results clearly showed that the rate of PG synthesis decreased with increased concentration of 4-MU4-deoxy-xyloside (Fig 1A), indicating that 4-MU4-deoxy-xyloside inhibited PG synthesis in a dose-dependent manner in lung primary fibroblasts. Indeed, at concentrations of 4-MU4-deoxy-xyloside ranging from 500 μM to 1 mM, PG synthesis was inhibited by 50% to 80% (Fig 1A).

To confirm the inhibitory effect of 4-MU4-deoxy-xyloside on PG-GAG synthesis, we analyzed its effect on endogenous GAG chains and on the formation of GAG chains primed with 4-MU-xyloside. Indeed, 4-MU-xyloside is used as a substrate by the β4GalT7 enzyme and further processed by glycosyltransferases of GAG biosynthetic pathway leading to the formation and secretion in the medium of GAG chains. Primary lung fibroblasts were cultured in medium containing radioactive Na_2_^35^SO_4_ in the absence (control) or presence of 4-MU4-deoxy-xyloside and 4-MU-xyloside. SDS-PAGE analysis of radiolabeled GAG chains clearly showed that 4-MU4-deoxy-xyloside decreased by about 50% the amount of endogenous GAG chains (Fig 1B and 1C). Similarly, 4-MU4-deoxy-xyloside reduced by about 48% the amount of GAG chains primed by 4-MU-xyloside (Fig 1B and 1C), indicating that it is able to inhibit synthesis of GAG chains.

### 4-MU4-deoxy-xyloside inhibits synthesis of both chondroitin- and heparan-sulfate PGs

**Fig 2:**
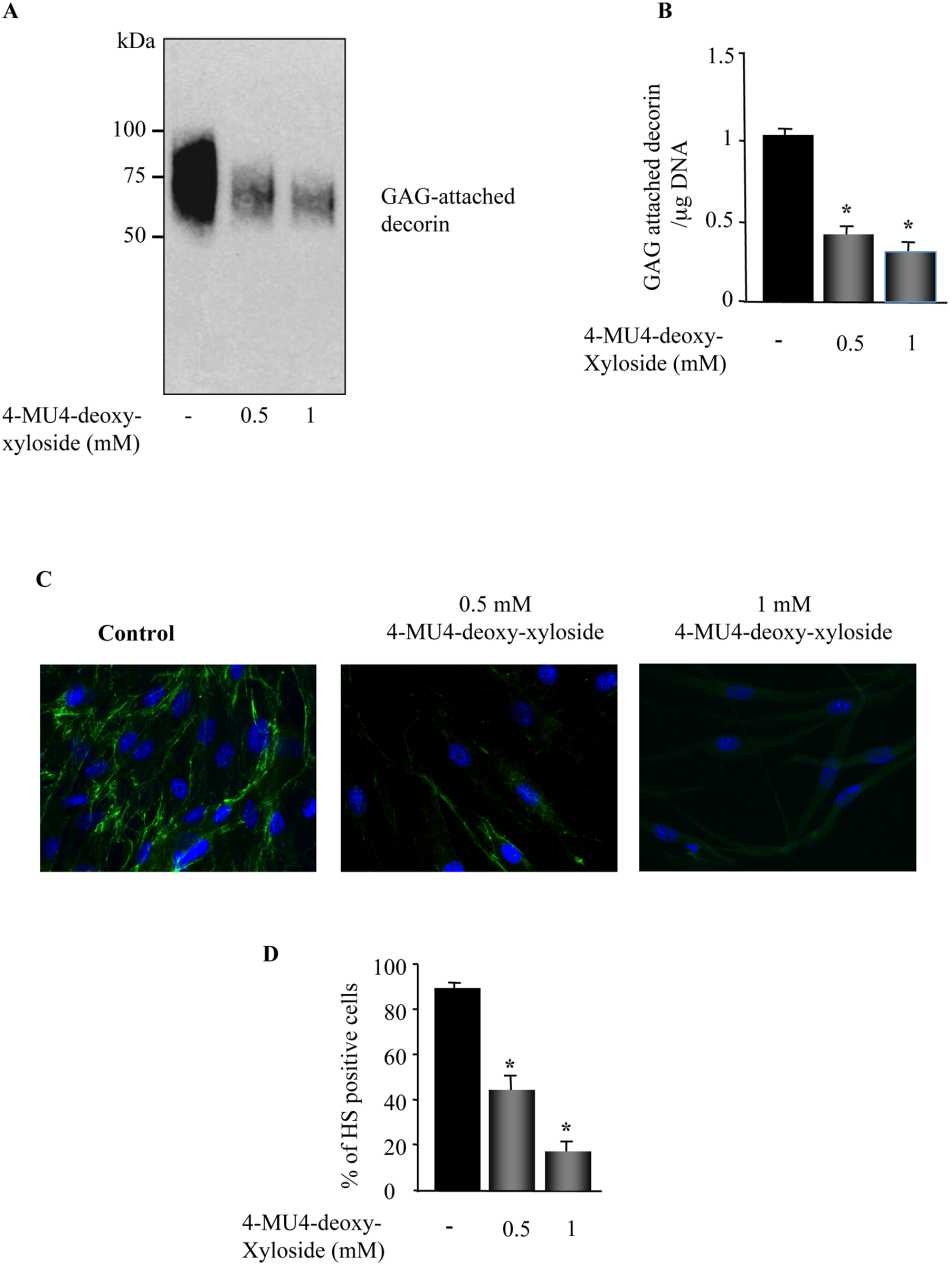
4-MU4-deoxy-xyloside inhibits the synthesis of both chondroitin- and heparin- sulphate PGs. Fibroblasts were treated with 500 μM or 1 mM of 4-MU4-deoxy-xyloside or with DMSO (control) for 24 h and (**A**) CS/DS-attached decorin produced was precipitated from culture medium by CPC and analyzed by Western blot using anti-decorin antibody. The absence of decorin core protein is due the use of CPC which selectively precipitate CS/DS-attached decorin. **(B)** Bar graph shows the amount of GAG-attached decorin normalized to DNA and relative to control (DMSO). Data are expressed as mean ± SD of three separate experiments; *P<0.05 versus control. (**C**) Lung fibroblasts were analyzed for the expression of cell surface HS-attached PGs by immunofluorescence using 10E4 monoclonal anti-HS antibodies. The Nuclei were stained by Hoechst dye. Representative micrographs were observed using inverted microscope Leica DMI3000 B (Leica Microsystems, Germany). The experiment was repeated twice. **(D)** The bar graph represented quantification of fluorescent intensity of heparin-sulphate in absence and presence of 4-MU4-deoxy-xyloside (500 μM or 1 mM). Data are expressed as mean ± SD of several images per condition; *P<0.05 versus control.

Given that β4GalT7 catalyses a common step to the synthesis of both CS- and HS-PGs, we examined whether 4-MU4-deoxy-xyloside inhibits the synthesis of both PG types. We first analyzed the effect of 4-MU4-deoxy-xyloside on the synthesis of decorin, a CS/DS-attached PG secreted in large amount by human skin primary fibroblasts. To this end, skin primary fibroblast cells were grown in the absence (control) and presence of 4-MU4-deoxy-xyloside and GAG-attached PGs were isolated from culture medium by CPC precipitation method and analyzed by Western blot using anti-decorin specific antibodies. The results clearly showed that 4-MU4-deoxy-xyloside inhibited, in a dose dependent manner, the synthesis of decorin (Fig 2A). As shown in Fig 2B, 4-MU4-deoxy-xyloside reduced by about 60% and 70% the amount of decorin at 500 μM and 1 mM, respectively.

We next analyzed the effect of 4-MU4-deoxy-xyloside on the synthesis of HS-PGs by analysing the expression of cell surface HS-attached PGs by indirect immunofluorescence using 10E4 anti-HS monoclonal antibodies, which are commonly used to detect HS chains of PGs [20]. Prominent staining of the cell membrane was observed in non-treated fibroblasts cells, whereas only low signal could be detected in 4-MU4-deoxy-xyloside-treated cells (Fig 2C). Fluorescent intensity quantification indicated that 4-MU4-deoxy-xyloside-treated cells exhibited a decrease of about 58% and 85% at 500 μM and 1 mM, respectively compared to control (Fig 2D), indicating that 4-MU4-deoxy-xyloside strongly reduced the synthesis of HS-GAG chains of PGs. Altogether, these results demonstrated that targeting the β4GalT7 enzyme by 4-MU4-deoxy-xyloside is an efficient strategy to inhibit the synthesis of PGs in lung primary fibroblats.

### 4-MU4-deoxy-xyloside inhibits TGF-β1-induced PG synthesis in primary lung fibroblasts

**Fig 3:**
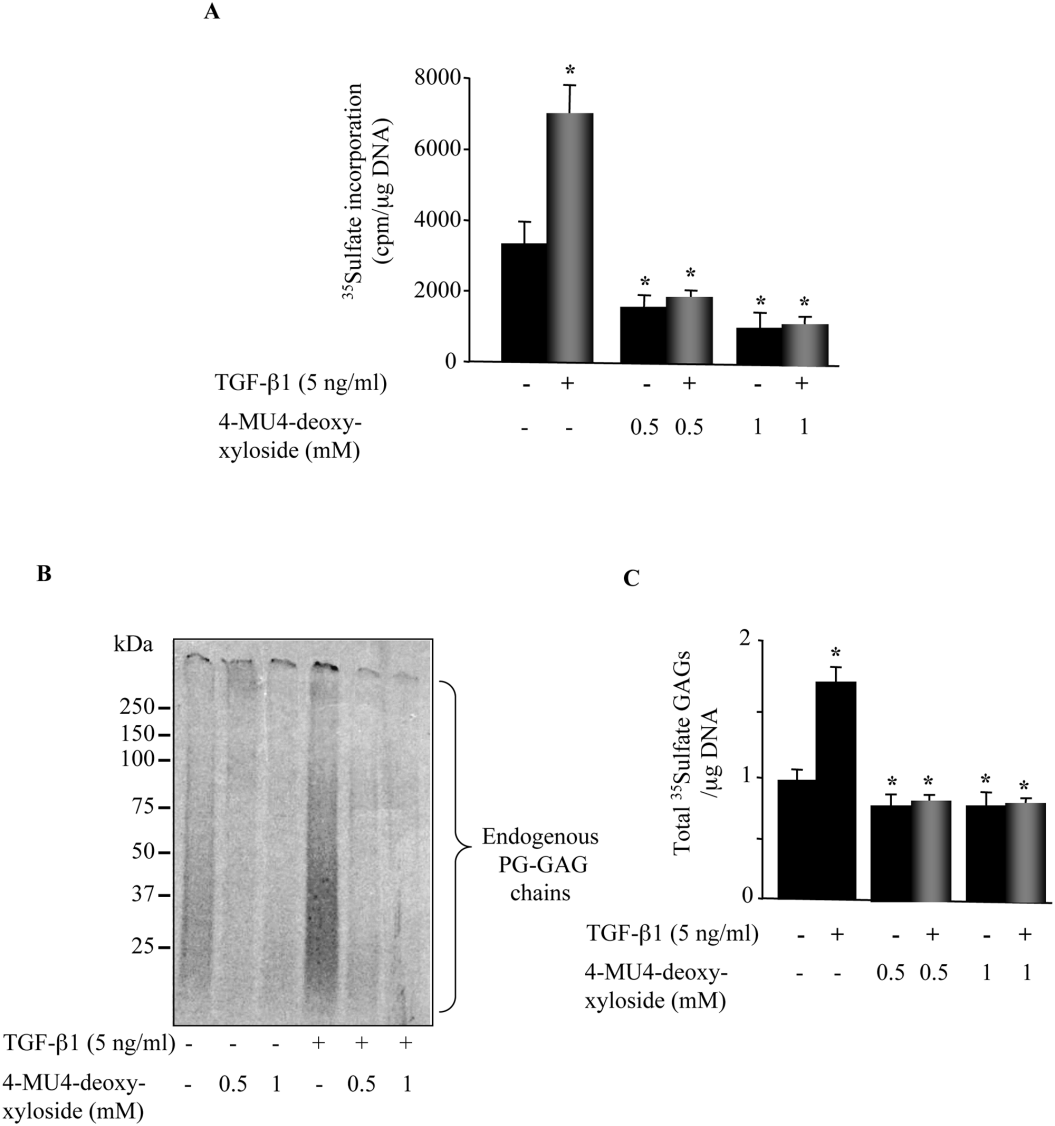
4-MU4-deoxy-xyloside antagonized TGF-β1-induced PG synthesis in lung fibroblasts. Cells were treated with 500 μM or 1 mM of 4-MU4-deoxy-xyloside or DMSO (control) and/or TGF-β1 (5 ng/ml) for 24 h and (**A**) the level of PG synthesis was measured by the ^35^Sulfate incorporation for the last 6 h of the treatment. The radioactivity associated with GAGs was evaluated by liquid scintillation counting after CPC precipitation and DNA normalization. Data are expressed as mean ± SD of three separate experiments; *P<0.05 versus control. (**B**) GAG chains were radiolabelled by ^35^Sulfate incorporation and isolated by CPC method at 24 h after the treatment and analyzed by SDS-PAGE then visualized by autoradiography. Bar graph shows the amount of ^35^Sulfate GAGs normalized to DNA and relative to control. Data are expressed as mean ± SD of three separate experiments; *P<0.05 versus control.

It has been shown that TGF-β1 is a major cytokine associated with fibrosis. TGF-β1 induces the synthesis of matrix proteins, particularly PGs leading to increased accumulation in the ECM, a process which is associated with fibrosis. Therefore, inhibiting TGF-β1-induced PG synthesis and deposition is of importance for the treatment of fibrosis. To determine whether 4-MU4-deoxy-xyloside is able to inhibit TGF-β1-induced increase of PG synthesis, lung fibroblasts were treated with TGF-β1 in the presence and absence of 4MU4-deoxy-xyloside for 24 h and PG synthesis was measured during the last 6 h of the treatment by ^35^S-Sulfation incorporation method. The results showed that treatment with TGF-β1 stimulated by about 2-fold the synthesis of PGs, compared to non-treated cells whereas, 4-MU4-deoxy-xyloside inhibited PG synthesis by about 2.2-fold and 3.8-fold at 0.5mM and 1mM, respectively (Fig 3A). However, in the presence of 4MU4-deoxy-xyloside, treatment with TGF-β1 did not induce any significant increase in PG synthesis, a rather decrease in the synthesis was observed (Fig 3A).

To further analyse the effect of 4MU4-deoxy-xyloside on PG synthesis, GAG chains were metabolically radiolabelled by ^35^S-Sulfation incorporation method for 24 h and newly synthesized radiolabeled PG-GAG chains were analyzed by SDS-PAGE. As shown in Fig 3B and 3C, 4-MU4-deoxy-xyloside significantly reduced the amount of endogenous GAG chains at both 0.5 mM and 1mM, compared to control (Fig 3B and 3C). Interestingly, while TGF-β1 induced a significant increase (75%) in the amount of GAG chains compared to control (Fig 3B and 3C), no significant changes in GAG chain content was produced by the cytokine in the presence of 4-MU4-deoxy-xyloside, a rather decrease was observed compared to control (Fig 3B and 3C). This clearly indicated that 4-MU4-deoxy-xyloside was able to counteract TGF-β1-induced increase in PG synthesis in lung primary fibroblasts.

### 4-MU4-deoxy-xyloside antagonizes TGF-β1-mediated Smad signaling

**Fig 4:**
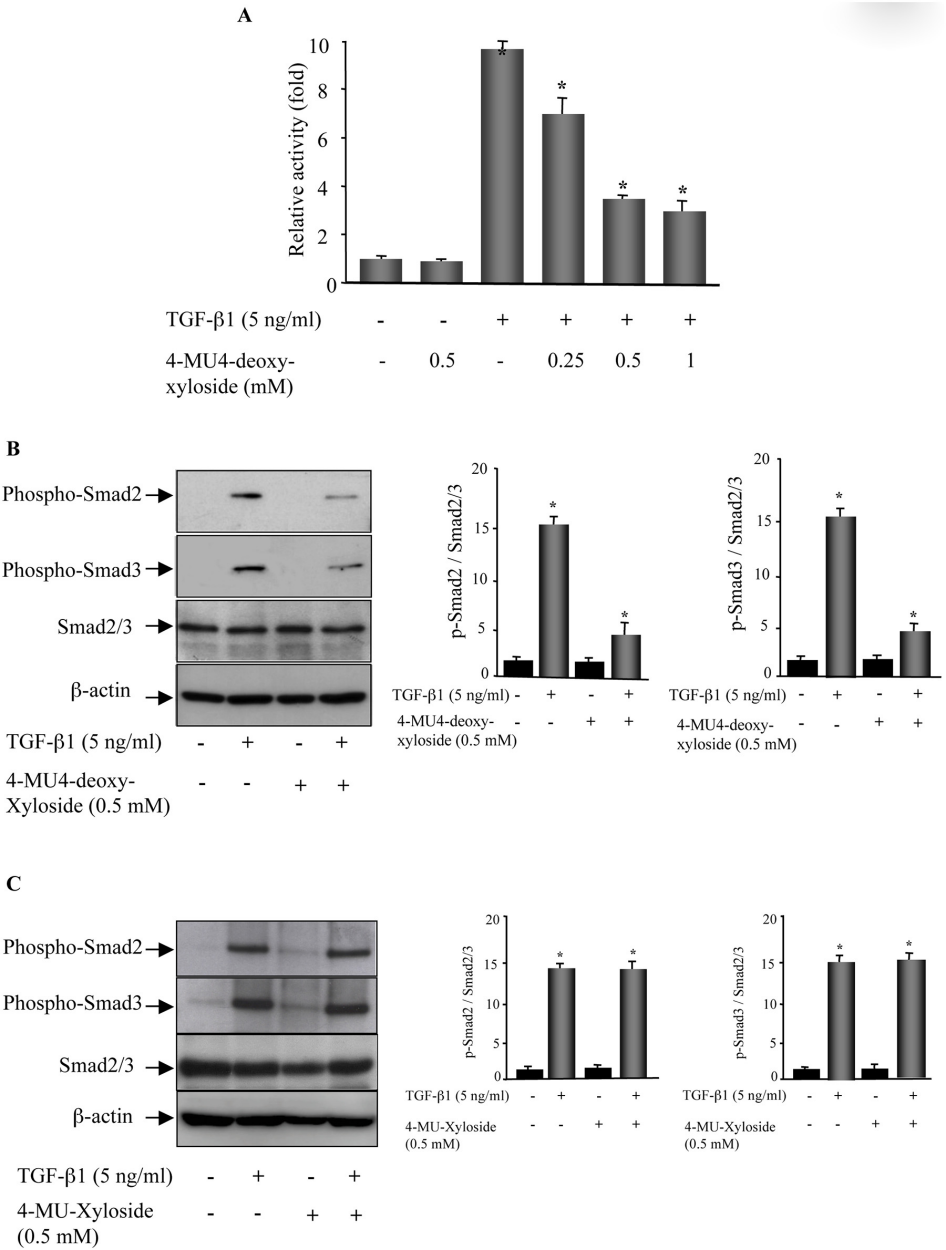
4-MU4-deoxy-xyloside blocks TGF-β1 signaling. (**A**) Lung fibroblasts were co-transfected with (CAGA)_12_-Lux reporter and pRL-TK (Renilla reporter) constructs and were incubated with DMSO (control) or with various concentrations of 4-MU4-deoxy-xyloside in the presence and absence TGF-β1 (5 ng/ml) for 24 h. The induction of the (CAGA)_12_-Lux reporter was measured by luciferase assay. Luciferase activities were normalized to pRL-TK vector activity. Data are expressed as mean ± SD of three separate experiments; *P<0.05 versus control. **(B)** Cells were incubated with 500 μM of 4-MU4-deoxy-xyloside or **(C)** with 500 μM of 4-MU-xyloside for 24 h then stimulated or not with TGF-β1 (5 ng/ml) for 3 h and assayed by Western blot to assess the levels of phosphorylation of Smad2 and Smad3. Bar graphs show the expression of phospho-Smad2 and phospho-Smad3 relative to total Smad2/3 level normalized to β-actin and expressed relative to control lysates. Data are expressed as mean ± SD of three separate experiments; *P<0.05 versus control.

It is well known that PGs play a critical role in the regulation of many ligand-mediated signalisation [21], therefore we hypothesized that 4-MU4-deoxy-xyloside treatment may impair TGF-β1 signaling in fibroblasts. To evaluate the impact of 4-MU4-deoxy-xyloside on TGF-β1 signaling, we used a (CAGA)_12_-Lux reporter which contains 12 copies of the Smad-binding site. As shown in Fig 4A, treatment of lung fibroblasts with TGF-β1 stimulated by about 10-fold the reporter activity, however this activation was strongly reduced in a dose dependent manner by 4-MU4-deoxy-xyloside (Fig 4A), indicating that 4-MU4-deoxy-xyloside antagonized the TGF-β1 signaling in primary lung fibroblasts. To further explore the intracellular signal transduction mechanism, we examined the effect of 4-MU4-deoxy-xyloside on the canonical Smad-dependent pathway. As shown in Fig 4B, 4-MU4-deoxy-xyloside significantly reduced TGF-β1-mediated phosphorylation of Smad2 and Smad3. However, this was not the case when 4-MU-xyloside was used (Fig 4C), indicating that 4-MU4-deoxy-xyloside, but not 4-MU-xyloside, repressed TGF-β1 canonical signaling pathway and suggested that soluble GAG chains primed by 4-MU-xyloside did not significantly affect TGF-β1 signaling.

### 4-MU4-deoxy-xyloside inhibits fibroblast proliferation

**Fig 5:**
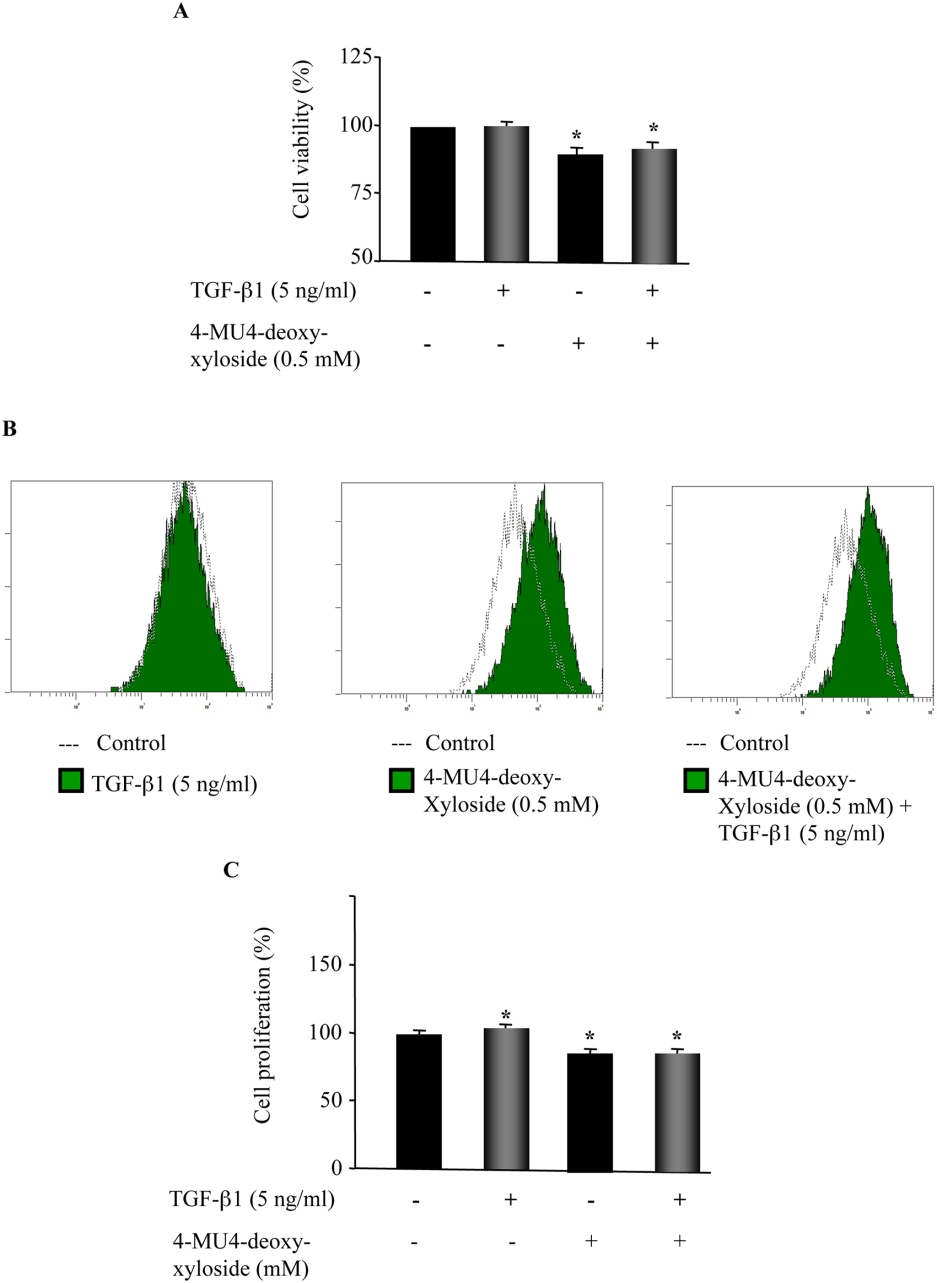
4-MU4-deoxy-xyloside reduces cell viability and inhibits cell proliferation. Lung fibroblasts were treated with DMSO (Control) or with 500 μM of 4-MU4-deoxy-xyloside and/or TGF-β1 (5 ng/ml) for 24 h. **(A)** Cell viability was measured by MTT assay. The relative cell viability (%) was expressed as a percentage relative to control cells. **(B)** Cell proliferation was analyzed by CSFC labelling. **(C)** Bar graph shows cell proliferation expressed as percentage relative to control cells (DMSO). Data are expressed as mean ± SD of three separate experiments. *P<0.05 versus control.

Because proliferation of fibroblasts/myofibroblasts and excessive production of matrix proteins are of major characteristics of pulmonary fibrosis, we then investigated the effect of 4-MU4-deoxy-xyloside on fibroblast proliferation. We first analyzed cell viability by MTT assay. As shown in Fig 5A, TGF-β1 stimulation did not impact the number of viable fibroblasts, whereas the cell viability was decreased by about 8% by 4-MU4-deoxy-xyloside treatment, compared to control. In the presence of TGF-β1, 4-MU4-deoxy-xyloside decreased cell viability by 3%, compared to TGF-β1-treated cells. These data indicated that 4-MU4-deoxy-xyloside is not cytotoxic. We next investigated the impact of 4-MU4-deoxy-xyloside on cell growth using CellTrace^™^ CFSE cell proliferation kit. TGF-β1 stimulation produced an increased of about 3% in cell proliferation, whereas treatment with 4-MU4-deoxy-xyloside induced a decrease of about 15% in fibroblast proliferation as indicated by delay in cell division measured by CFSE dye and flow cytometry (Fig 5B and 5C). In addition, 4-MU4-deoxy-xyloside was able to counteract the stimulatory effect of TGF-β1 on cell proliferation and maintain a significant decrease (14%) in cell proliferation even in the presence of TGF-β1 (Fig 5B and 5C).

### 4-MU4-deoxy-xyloside reduces collagen synthesis in primary lung fibroblasts

**Fig 6:**
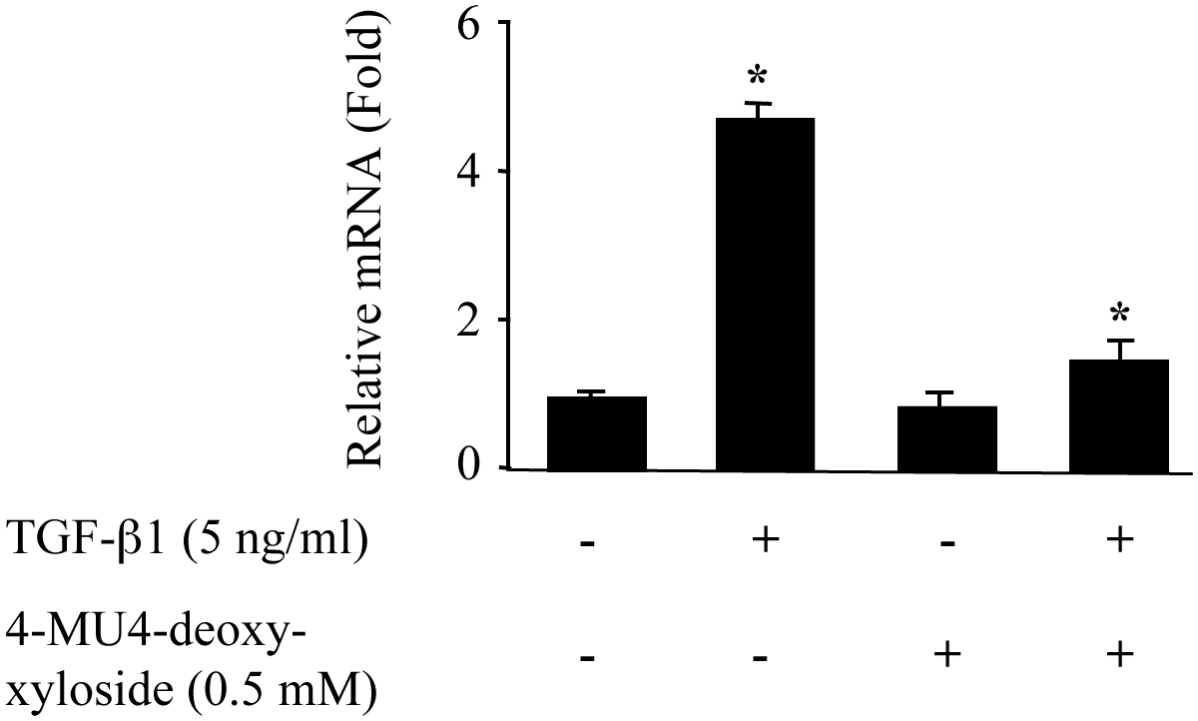
4-MU4-deoxy-xyloside reduced TGF-β1-induced increased expression of collagen type 1. Lung fibroblasts were treated with 500 μM of 4-MU4-deoxy-xyloside and/or TGF-β1 (5 ng/ml) for 6 h and the expression of endogenous TGF-β1was analyzed by qPCR. The relative expression was normalized with ribosomal protein S29. Data are expressed as mean ± SD of three separate experiments, (*P<0.05).

Fibrosis is a process associated with overproduction of collagen type I, which together with PGs constitute the major contributor of the ECM deposition in the lung. We showed above that 4-MU4-deoxy-xyloside counteracts TGF-β1-induced increase in PG synthesis, we next examined the effect of 4-MU4-deoxy-xyloside on TGF-β1-induced production of fibrotic matrix collagen type I. Treatment of the cells with TGF-β1 increased by 4.7-fold the expression of collagen type I (Fig 6). Interestingly, in the presence of 4-MU4-deoxy-xyloside, TGF-β1-induced expression of collagen I was reduced by 70% (Fig 6), suggesting an anti-fibrotic role of 4-MU4-deoxy-xyloside in counteracting TGF-β1-induced ECM production.

### 4-MU4-deoxy-xyloside prevents fibrogenic activation of lung primary fibroblasts

**Fig 7:**
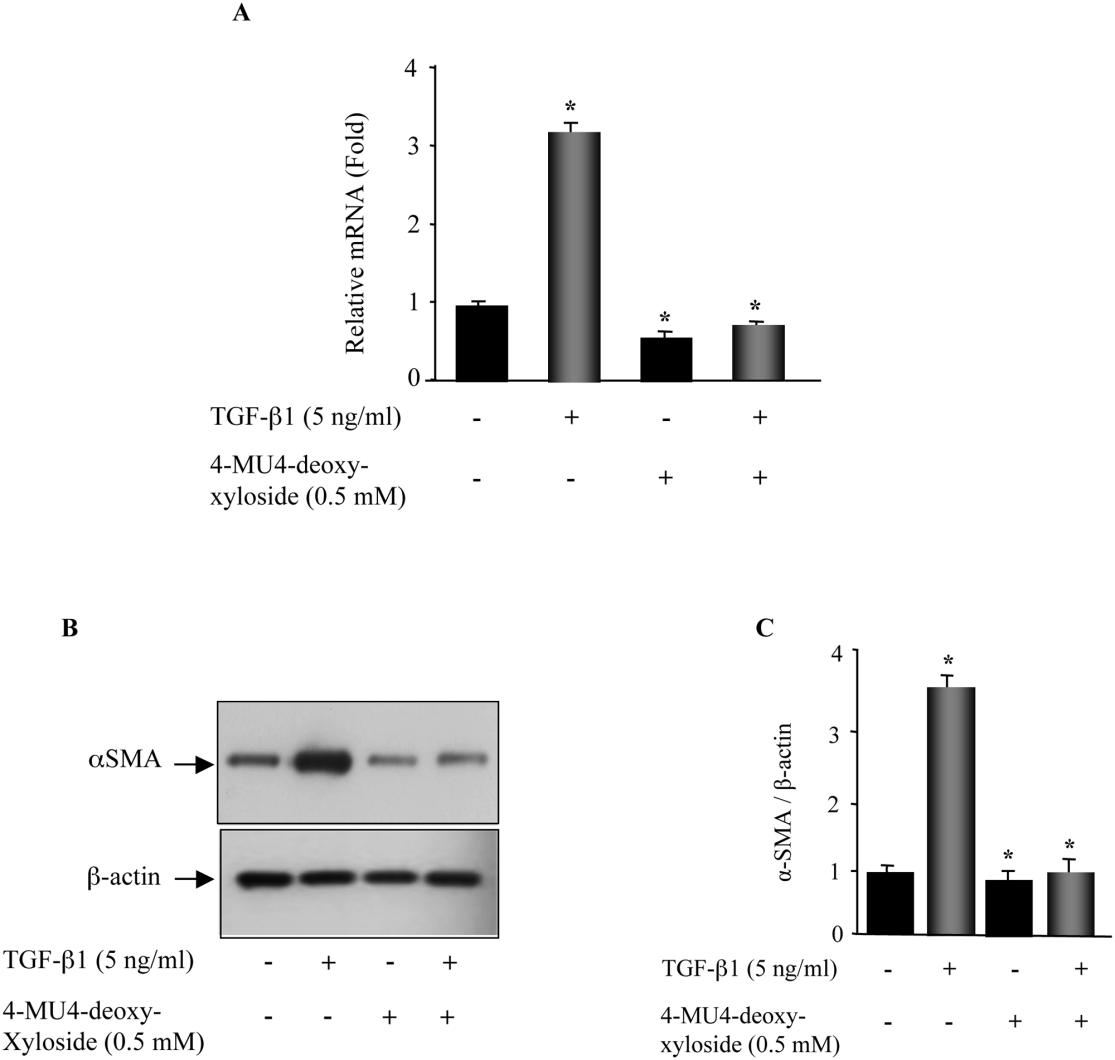
4-MU4-deoxy-xyloside counteracts TGF-β1-induced fibroblast-to-myofibroblast trans-differentiation. Lung fibroblasts were treated with 500 μM of 4-MU4-deoxy-xyloside and/or TGF-β1 (5 ng/ml) for 24 h and the expression of αSMA was analyzed by qPCR (**A**) and by Western Blot (**B**). The relative expression was normalized with ribosomal protein S29. Data are expressed as mean ± SD of three separate experiments; *P<0.05 versus control. For Western blot, whole cell lysates were analysed αSMA content. β-actin was used as a loading control. **(C)** Bar graph shows the expression of αSMA relative to β-actin level and expressed relative to control lysate. Data are expressed as mean ± SD of three separate experiments; *P<0.05 versus control.

In pulmonary fibrosis, TGF-β1 promotes transdifferentiation of quiescent fibroblasts into myofibroblasts which excessively produce ECM components [22, 23]. As 4-MU4-deoxy-xyloside was able to counteract TGF-β1 signaling, we therefore examined the effect of 4-MU4-deoxy-xyloside on TGF-β1-activation of fibroblasts by analysing the expression of α-smooth muscle actin (αSMA), a reliable marker of activation of fibroblasts. As shown in Fig 7A, qPCR analysis indicated a 3-fold induction in the expression of αSMA in TGF-β1-treated fibroblasts, compared to non-treated cells. However, in the presence of 4-MU4-deoxy-xyloside, TGF-β1 failed to induce the expression of αSMA. Similar results were obtained by Western blot (Fig 7B and 7C). This indicated that 4-MU4-deoxy-xyloside prevented fibroblast-to-myofibroblast trans-differentiation in response to TGF-β1.

### 4-MU4-deoxy-xyloside inhibited the autocrine expression of TGF-β1 in primary lung fibroblasts

**Fig 8:**
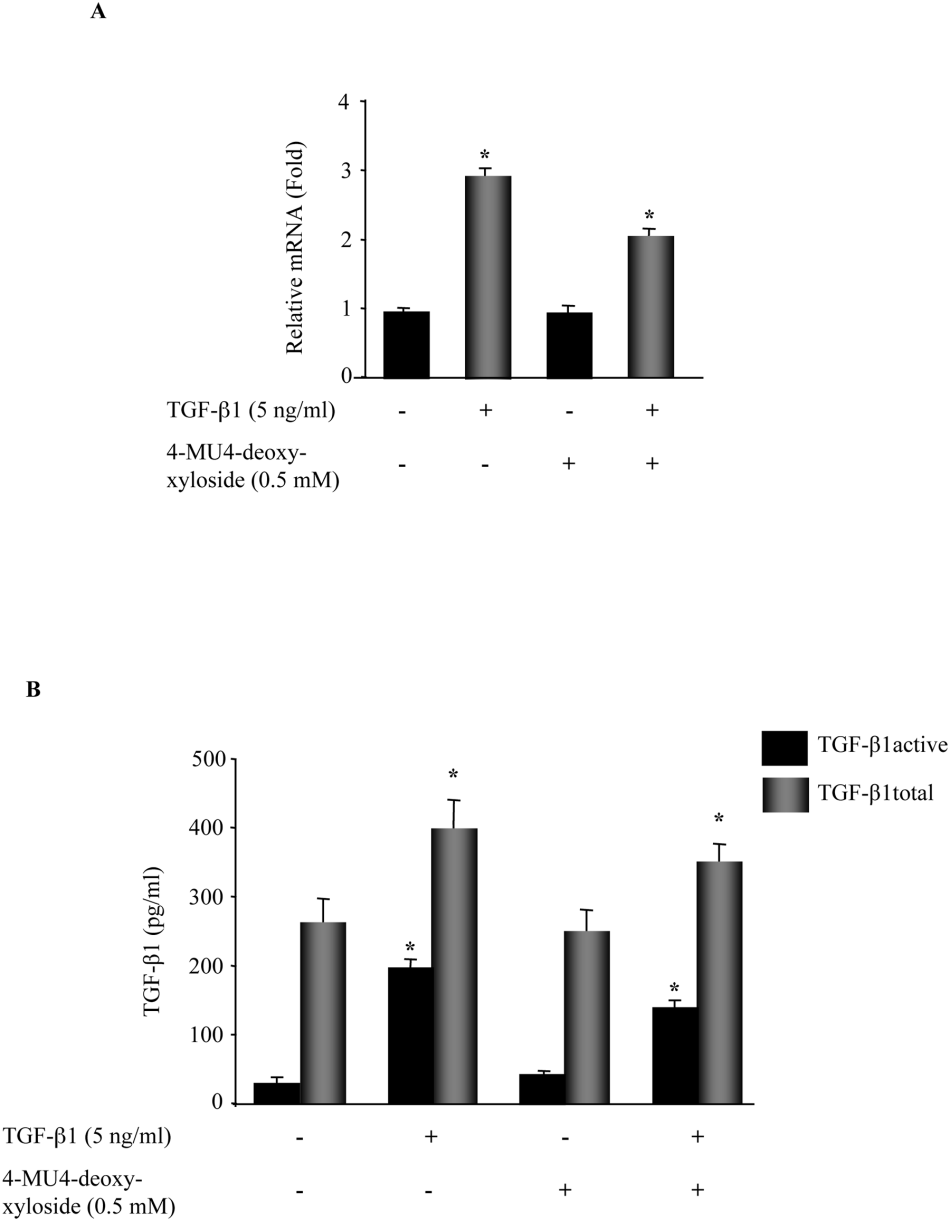
4-MU4-deoxy-xyloside reduced TGF-β1-induced increased expression and actvation of endogenous TGF-β1. **(A)** Lung fibroblasts were treated with 500 μM of 4-MU4-deoxy-xyloside and/or TGF-β1 (5 ng/ml) for 6 h and the expression of endogenous TGF-β1was analyzed by qPCR. The relative expression was normalized with ribosomal protein S29. Data are expressed as mean ± SD of three separate experiments, (*P<0.05). **(B)** Fibroblasts were treated with 500 μM of 4-MU4-deoxy-xyloside and/or TGF-β1 (5 ng/ml) for 24 h and the total and active TGF-β1 levels were measured by ELISA in cell culture supernatants as described in Materials and methods section.

It is well known that TGF-β1 is expressed in an autocrine manner [24], therefore we examined whether 4-MU4-deoxy-xyloside could impair the endogenous TGF-β1 expression. Evaluation of TGF-β1 expression in response to external TGF-β1-stimulation showed an increase of about 3-fold, indicating that TGF-β1 is expressed in an autocrine manner in lung primary fibroblasts (Fig 8A). However, in the presence of 4-MU4-deoxy-xyloside, the induction of the expression of endogenous TGF-β1 transcripts by TGF-β1 was reduced by 30% (Fig 8A), indicating that 4-MU4-deoxy-xyloside was able to counteract to some extent the autocrine expression of TGF-β1 in lung primary fibroblasts.

We next measured active and total TGF-β1 levels in culture cell supernatant by ELISA. As shown in Fig 8B, the active and total TGF-β1 levels in culture supernatant of control cells were 28 ± 7 pg/ml and 287 ± 25 pg/ml, respectively, indicating that the total TGF-β1 level was about 10-fold higher than the active TGF-β1 level. Similar results were obtained when cells were treated with 4-MU4-deoxy-xyloside. Treatment with TGF-β1 increased by 1.5-fold and 7-fold the levels of total and active TGF-β1, respectively compared to control. Noteworthy, the total TGF-β1 level was only 2-fold higher than the active TGF-β1 level, indicating that stimulation of fibroblasts with TGF-β1 enhanced the secretion and activation of the cytokine. Interestingly, treatment with 4-MU4-deoxy-xyloside reduced TGF-β1-induced increased levels of total and active TGF-β1 by 20% and 30% (Fig 8B), respectively indicating that 4-MU4-deoxy-xyloside significantly reduced the activation and secretion of TGF-β1 in response to external TGF-β1 stimulation. Altogether, these results indicated that 4-MU4-deoxy-xyloside impairs TGF-β1-mediated activity in lung primary fibroblasts and pointed out the requirement for PG-GAGs for a profibrotic phenotype in lung fibroblast cells responding to TGF-β1.

### Silencing of β4GalT7 impaired TGF-β1-induced effects in lung fibroblasts

**Fig 9:**
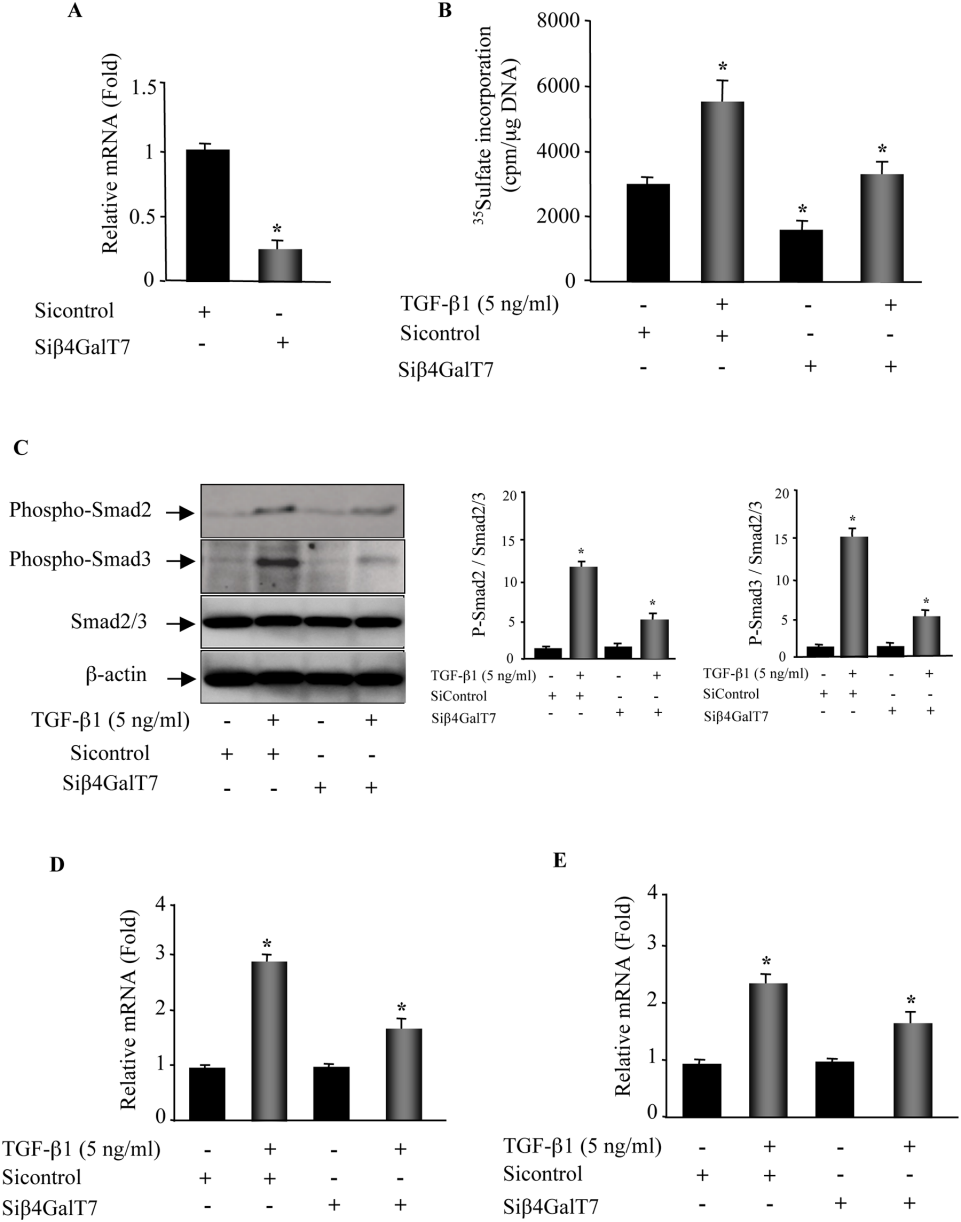
Silencing of β4GalT7 antagonized TGF-β1-induced expression of αSMA and TGFβ-1 and activation of Smad signaling pathway. (**A**) Lung fibroblasts were transfected with siRNA for β4GalT7 or siRNA control for 48 h and the expression of β4GalT7 was analysed by qPCR. (**B**) Cells transfected with Siβ4GalT7 or Sicontrol for 48 h were stimulated or not with TGF-β1 (5 ng/ml) for 24 h and the level of PG synthesis was measured by ^35^Sulfate incorporation during the last 6h of TGF-β1 treatment. The radioactivity associated with GAGs was evaluated by liquid scintillation counting after CPC precipitation and normalized to DNA content. (**C**) Cells were transfected with Siβ4GalT7 or Sicontrol for 48 h then stimulated or not with TGF-β1 (5 ng/ml) for 3 h and assayed by Western blot to assess the levels of phosphorylation of Smad2 and Smad3. β-actin was used as a loading control. Bar graphs show the expression of phospho-Smad2 and phospho-Smad3 relative to total Smad2/3 level normalized to β-actin and expressed relative to control lysates. Data are expressed as mean ± SD of three separate experiments; *P<0.05 versus control. (**D**) and (**E**), the expression of αSMA and of endogenous TGF-β1 were analysed by qPCR after 6 h of TGF-β1 treatment in fibroblasts transfected with Siβ4GalT7 and Sicontrol. The relative expression was normalized with ribosomal protein S29. Data are expressed as mean ± SD of three separate experiments; *P<0.05 versus control.

To determine whether the effects produced by 4-MU4-deoxy-xyloside were specific to the inhibition of β4GalT7 enzyme activity, the expression of β4GalT7 was silenced by siRNA in lung fibroblasts and TGF-β1-induced effects were investigated. The knockdown of β4GalT7 decreased by about 75% the expression of β4GalT7, compared to control (Fig 9A) and reduced by 50% the level of PG synthesis (Fig 9B), indicating that silencing of β4GalT7 significantly impaired the synthesis of PGs. As observed for 4-MU4-deoxy-xyloside, silencing of β4GalT7 in lung fibroblasts counteracted TGF-β1-induced increase in PG synthesis. Indeed, TGF-β1 increased the level of PG synthesis by about 2-fold in control cells, whereas in β4GalT7 knockdown cells the increase was only of about 14% (Fig 9B). Furthermore, investigation of the effect of β4GalT7 knockdown on TGF-β1 canonical signaling indicated that, as observed for 4-MU4-deoxy-xyloside, silencing of β4GalT7 antagonized TGF-β1-induced activation of Smad signaling pathway (Fig 9C). Moreover, similarly to 4-MU4-deoxy-xyloside, knockdown of β4GalT7 reduced TGF-β1-stimulated increase in the expression of αSMA from 3-fold to 1.5-fold (Fig 9D) and of endogenous TGF-β1 from 2.6-fold to 1.8-fold (Fig 9E), therefore antagonizing fibroblast-to-myofibroblast trans-differentiation and the autocrine expression of TGF-β1 induced by the cytokine. Altogether, these data showed that knockdown of β4GalT7 led to similar effects to those produced by 4-MU4-deoxy-xyloside in response to TGF-β1 stimulation in lung primary fibroblasts, indicating that the effects produced by 4-MU4-deoxy-xyloside were specific to the inhibition of β4GalT7 enzyme.

## Discussion

Idiopathic pulmonary fibrosis is a progressive and fatal disease with no effective therapies. Lung transplantation is the only viable therapy for end stage organ failure [25, 26], therefore IPF represents a major unmet medical need for which novel therapeutic approaches are immediately required. Though inflammation has usually been considered as the major factor of lung fibrosis, emerging evidence [27] indicates that dysregulation of the ECM metabolism plays a critical role in pathogenesis of lung fibrosis, supporting studies on matrix production and deposition. One of the features of lung fibrosis is the accumulation of collagen and PGs in the ECM. The augmented levels of PGs in fibrosis is a part of the exacerbated accumulation of ECM constituents that is characteristic of the fibrotic process and as a cell response to increases in in the levels of profibrotic growth factors such as TGF-β. It is well known that PGs, owing to their ability to mediate the effects of several ligands, play a major role not only in ECM architecture but also in the regulation of several key cellular events such as proliferation and differentiation. Therefore, PG based therapies for IPF may be beneficial in clinical settings.

Earlier we have reported changes in glycosyltransferases as the underlying mechanism for abnormal PG biosynthetic activity in IPF, as well as the potential effect of TGF-β1 on PG-GAG biosynthetic machinery in pulmonary fibrosis [9]. Here we demonstrated for the first time that treatment of lung primary fibroblasts with 4-MU4-deoxy-xyloside, an inhibitor of β4GalT7, led to a strong decrease in PG synthesis. We demonstrated that 4-MU4-deoxy-xyloside inhibited the production and accumulation of both CS/DS-PGs and HS-PGs in primary fibroblasts. These observations indicated that 4-MU4-deoxy-xyloside could play an antifibrotic role in the formation of lung matrix by negatively regulating the synthesis of PGs.

TGF-β1 has been implicated as a key factor in the pathogenesis of fibrosis with stimulatory effects, among which are the increase of collagens, PGs, fibrotic growth factors [28]. Based on these reports, we investigated whether 4-MU4-deoxy-xyloside was able to prevent TGF-β1-induced increased PG synthesis in lung primary fibroblasts. Our results clearly showed that 4-MU4-deoxy-xyloside was able to counteract the increase in PG synthesis produced by the cytokine, indicating that 4-MU4-deoxy-xyloside is effective in preventing TGF-β1-induced production of PGs in lung primary fibroblasts.

Given that PGs are co-distributed with collagens in the ECM, and that small PGs such as decorin are critical for collagen deposition and integration into the ECM, inhibition of PG synthesis by 4-MU4-deoxy-xyloside may influence the assembly of the ECM fibrils during lung fibrosis. Therefore, we examined whether 4-MU4-deoxy-xyloside affects the expression of other ECM proteins induced by TGF-β1 such as collagen type I, in addition to PG-GAGs. It was noted that fibroblasts cultured in the presence of 4-MU4-deoxy-xyloside alone produced no change in collagen I expression level. In contrast, 4-MU4-deoxy-xyloside inhibited the ability of TGF-β1 to increase collagen I transcripts. These findings indicate that collagen deposition into the ECM could be reduced in 4-MU4-deoxy-xyloside-treated fibroblasts. Previous studies have demonstrated that Smads act as crucial mediators for collagen regulation by TGF-β1 *in vitro*. It has been shown that transient overexpression of Smad3 in normal dermal fibroblasts mirrored the effect of TGF-β1 on collagen gene transcription [29], therefore inhibiting cellular Smad3 expression and/or phosphorylation would block type I collagen transcription by TGF-β1. Here we found that 4-MU4-deoxy-xyloside blocked the phosphorylation of Smad2/3, therefore a potential benefit of Smad2/3 inactivation by 4-MU4-deoxy-xyloside may favour inhibition of TGF-β1-induced collagen expression during fibrogenesis.

Increasing evidence points to the importance of fibroblast-to-myofibroblast trans-differentiation in fibrosis. Indeed, myofibroblasts express αSMA and regulate tissue regeneration, ECM production, cell-cell interaction and wound contraction [30]. TGF-β1 has been widely recognized as a key fibrogenic cytokine and has been demonstrated to activate fibroblasts differentiation into myofibroblasts *in vitro* and *in vivo* [31, 32] and to upregulate αSMA expression through the enhancement of Smad phosphorylation [33, 34]. Interestingly, we showed here that treatment of lung primary fibroblasts with 4-MU4-deoxy-xyloside prevented myofibroblast phenotype induced by TGF-β1 as evidenced by failure of TGF-β1 to induce the myofibroblast marker αSMA. These findings indicate that reduction of myofibroblast phenotype by 4-MU4-deoxy-xyloside might improve wound-healing and other features of tissue repair.

TGF-β1 is expressed in an autocrine manner [24] and understanding both active and total TGF-β1 levels would provide us with further insight into how TGF-β1 is involved in physiological and pathological conditions such as fibrosis. Our results demonstrated that the majority of TGF-β1 present in primary lung fibroblasts exists as a latent form with only a fraction being biologically active (10%). However, stimulation with TGF-β1 not only increased the secretion of TGF-β1, but also enhanced its activation as the active form represents up to 50% of the total levels of TGF-β1. Interestingly, treatment with 4-MU4-deoxy-xyloside reduced both secretion and activation of the cytokine, in response to external TGF-β1 stimulation.

To gain insight into the mechanism by which 4-MU4-deoxy-xyloside antagonized the effects of TGF-β1 in lung primary fibroblasts, we examined its effect on TGF-β signaling pathway. Once activated, TGF-β binds to TGFβ type II receptor leading to the recruitment of the TGFβ type I receptor which induces the phosphorylation of the downstream targets Smad2 and Smad3. TGF-β has another cell surface receptor, the HSPG β-glycan or TGF-β receptor III. This HSPG has two independent high-affinity binding domains for TGF-β [35], thus it can present TGF-β to the type II receptor to activate the canonical Smad signaling pathway [36]. It is well known that Smad signal transduction pathways are crucial in mediating TGF-β1 response in fibroblasts [37, 38]. Studies about fibroblasts derived from embryos null for either Smad3 or Smad2 revealed that TGF-β-regulated genes dependent on either Smad2 or Smad3 or both [39]. Investigation of the effect of 4-MU4-deoxy-xyloside on TGF-β1 phosphorylation of Smad2/3 and the expression of total Smad2/3 in lung primary fibroblasts showed that 4-MU4-deoxy-xyloside strongly attenuated the phosphorylation of Smad2 and Smad3 induced by TGF-β1. However, treatment with 4-MU-xyloside did not affected TGF-β1-induced Smad activation. Given that 4-MU-xyloside acts as a competitive acceptor with the endogenous core protein of PG for CS synthesis and primes soluble GAG chains of mainly CS-type, this suggests that neither free CS-GAGs produced nor the competition with endogenous PG core proteins significantly alter TGF-β1 signaling.

To ascertain that the effects produced by 4-MU4-deoxy-xyloside in lung fibroblast cells were due to the specific inhibition of the β4GalT7, the expression of this enzyme was inhibited by siRNA in lung primary fibroblasts. The knockdown of β4GalT7 impaired TGF-β1-induced stimulation of PG synthesis, Smad signaling and, expression of αSMA and TGF-β1, thus leading to similar effects to those produced by 4-MU4-deoxy-xyloside and therefore, attributing the effects of 4-MU4-deoxy-xyloside to inhibition of the β4GalT7 enzyme.

The mechanism by which 4-MU4-deoxy-xyloside antagonized TGF-β1 signaling is unknown, however it has been shown that myoblasts require decorin for a full TGF-β cell response, by a mechanism that is dependent on the giant receptor lipoprotein receptor-related protein (LRP1) (74–75). We showed here that treatment of primary fibroblasts with 4-MU4-deoxy-xyloside led to strong decrease of PG synthesis in general and of decorin in particular. However, to determine whether this mechanism is relevant to lung fibroblasts needs further investigations. Noteworthy, TGF-β receptor type III or βglycan is an HS-PG that function as a TGF-β co-receptor by binding the TGF-β and presenting the ligand to the TGF-β type II receptor. Once bound to ligand, TGF-β type II receptor recruits and trans-phosphorylates the TGF-β type I receptor, activating its kinase function and leading to the phosphorylation of Smad2/3 [36]. Therefore, inhibition of HS-GAG chains synthesis may alter the function or the stability of this HSPG receptor and impairs TGF-β1 signaling.

To conclude, our present results demonstrate that 4-MU4-deoxy-xyloside affects fibroblast biosynthetic function and differentiation and reduced TGF-β1 auto-production which could affect in situ lung fibrotic repair. Collectively, our data suggest that 4-MU4-deoxy-xyloside holds therapeutic benefits as antifibrotic agent that may be valuable in pulmonary fibrosis.
